# Mobility and Retention of Rare Earth Elements in Porous Media

**DOI:** 10.1021/acsomega.2c01180

**Published:** 2022-06-02

**Authors:** Nitai Amiel, Ishai Dror, Brian Berkowitz

**Affiliations:** Department of Earth and Planetary Sciences, Weizmann Institute of Science, Rehovot 7610001, Israel

## Abstract

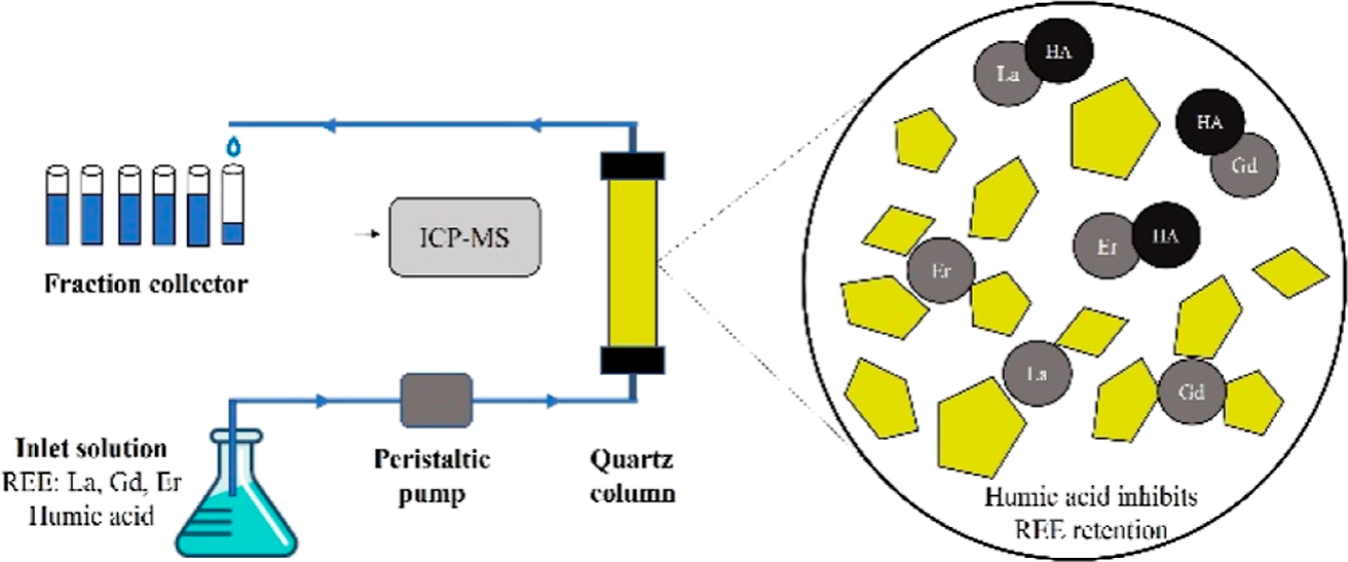

There is growing concern that rare earth elements (REEs) will become emerging soil–water contaminants because of their increased use in new technologies and products, which may lead to unavoidable release to the environment. To better understand the environmental behavior of REEs, a comprehensive set of adsorption and column transport experiments was conducted in quartz sand media. The retention and mobility of three representative REEs (La, Gd, and Er) were studied in the presence and absence of humic acid (HA; 5, 20, and 50 mg L^–1^) and under a range of pH conditions (5–8). Results show that REE mobility and retention are controlled by the amount of REE–HA complexes formed in a solution, which increases with increasing HA concentrations and solution pH. Gadolinium is the most mobile among the representative REEs, followed by Er and La, corresponding to the amount of (calculated) REE–HA complexes. Increasing HA concentrations in the REE solution inhibits REE retention in both the batch adsorption and column experiments. The same retardation trend was observed for lower HA concentrations (Gd > Er > La). In a fixed HA concentration, HA and REE adsorption decrease simultaneously as the solution pH increases, indicating the co-adsorption of REEs and HA on the sand. Scanning electron microscopy detection of elongated regions attached to the sand, where high REE and carbon (HA) concentrations were measured, further suggests the co-adsorption of REE–HA complexes. Modeling the column experiments shows that the time-dependent attachment is dominant at high HA concentrations, while at lower HA concentrations, both the time-dependent and spontaneous attachments play equal roles. These results provide a quantitative characterization of REE retention and mobility in sand media.

## 1 Introduction

Rare earth elements (REEs) are defined as the lanthanide series elements, together with yttrium and scandium. The REEs are divided into three overlapping groups: (1) light REEs (LREEs; La–Sm), (2) middle REEs (MREEs; Sm–Dy), and (3) heavy REEs (HREEs; Er–Lu and Y).^1,2^ REEs have become critical elements in many emerging technologies, with applications to, for example, water treatment, metal alloys, magnets, nuclear reactors, catalysts, and medicine.^2,3^ Thus, the ever-increasing use of REEs may lead to concerns about a significant release to the environment—during production and/or following use or disposal—that could ultimately lead to soil and water pollution, accumulation in plants, and toxic exposure to living organisms.^4−7^

REEs share common physiochemical properties, such as their almost uniform trivalent oxidation state. The ionic radius of the REEs gradually decreases through the lanthanide series due to the gradual filling of the “4f” orbital, which imperfectly shields the nuclear charge, known as the “lanthanide contraction”.^2^ As a result, the “lanthanide contraction” causes ligand complexation affinity to increase across the lanthanide series.^8^

The hydrogeochemical behavior of REEs is derived from their aquatic speciation.^9,10^ REE speciation in aquatic systems varies among free trivalent ions, organic and inorganic complexes, and colloidal transport. Changing the environmental conditions significantly affects REE speciation due to the high sensitivity of REEs even to small changes in the solution chemistry.^9,11,12^ Field studies have shown that REE speciation in various aquatic systems is dominated by the colloidal fraction (sizes of 1 nm to 1 μm), which includes humic substances [humic acid (HA) and fulvic acid], carbonates, clays, and oxyhydroxides, among others.^8,13−17^ HA colloids, which are macromolecules with a high variety of functional groups that can bind a large number of metal ions, have been shown to control REE mobility and distribution in both natural aquatic systems and in laboratory experiments.^16−22^ The HA concentration in natural aquatic systems varies from 0.5–1 mg L^–1^ for groundwater, 1–10 mg L^–1^ for lakes and rivers, and 10–50 mg L^–1^ for wetlands.^23^

The complexation of REE to HA and the effect of HA on the environmental behavior of REEs have been intensively studied. Such processes were found to be affected strongly by chemical parameters such as pH,^21,24−27^ HA concentrations, metal loading,^16,21,28−30^ ionic strength,^31^ the presence of other complexing agents,^9,10,32^ the presence of Fe–Mn oxyhydroxides,^33−35^ and the presence of nanoparticles^36,37^ in the solution. Generally, the amount of REE–HA complexes increases as the pH and the metal loading (REE/HA ratio) increase.

Although the aquatic behavior of REEs has been studied widely, mainly through in situ measurements and batch experiments,^20,35,36,38,39^ only a few studies have examined the effect of changes in the leading physicochemical parameters on the mobility of REE–HA complexes in porous media. The majority of REE column experiments involved trivalent europium (Eu^+3^) because of its use as a homolog for trivalent radioactive actinides.^31,40−42^

Mobility experiments of REE mixtures with HA are scarce,^16,37,43^ as none of the studies focused on how changes in HA concentrations and solution pH affect REE transport. Brewer et al.^37^ studied REE transport through sand and soil columns under a wide range of conditions; in particular, complete REE adsorption was found in soil column experiments in the presence of 5 mg L^–1^ HA in the solution. As a consequence, the effects of key chemical parameters on the mobility and retention of the different REEs in the presence of HA in porous media are poorly understood.

In this context, we conducted a series of column experiments accompanied by batch experiments and transport modeling. The main objectives of this study are to examine REE retention and mobility in quartz sand media under (i) different natural pH conditions (5–8) and (ii) with or without the addition of HA (0, 5, 20, and 50 mg L^–1^) to study differences in the transport behavior of the selected REEs (La, Gd, and Er).

## 2 Materials and Methods

### 2.1 Reagents

Lanthanum (III) nitrate hexahydrate 99.99%, gadolinium (III) nitrate hydrate 99.9%, erbium (III) nitrate pentahydrate 99.9%, sodium bromide (NaBr ≥ 99.5%), nitric acid (HNO3 70%), and HA sodium salt were purchased from Sigma-Aldrich.

Quartz sand (mesh size 30/40) was purchased from UNIMIN, USA. The sand was immersed in 5% nitric acid for 24 h, followed by a wash with double deionized water (DDW, 18.2 > MΩ cm^–1^). The washed sand was then dried in an oven at 105 °C for 24 h prior to the experiments.

All the inlet solutions were prepared from REE and HA stock solutions. An REE stock solution with a concentration of 50 mg L^–1^ of La, Gd, Er, and Br. An HA stock solution was prepared by dissolving 1 g of Aldrich HA in 1 L of DDW at pH 10 (adjusted using 0.1 M NaOH). The REE stock solution was diluted X50 and mixed with DDW and HA, which was further diluted to reach the required concentration. All the solutions were prepared using DDW.

### 2.2 Speciation Calculation

REE speciation in the adsorption and column experiment inlet solutions were calculated using the Stockholm Humic Model (SHM), integrated into Visual MINTEQ (Version 3.1).^44^ The experimental conditions of the entire set of column and adsorption experiments, and the eluted REE recoveries, are detailed in Table S2. The eluted phase recoveries were calculated by integrating the REE concentrations from the experimental breakthrough curves (BTCs).

The SHM is a discrete ligand model in which HA is assumed to have eight proton-binding sites with distinct acid–base characteristics. Additional information on the SHM is discussed in Gustafsson.^45,46^ Two parameters are needed to calculate REE speciation in the SHM; the intrinsic equilibrium constant for bidentate complexation (log K_Mb_) and the distribution term that modifies the strength of complexation sites (ΔLK_2_). As Gustafsson^45^ demonstrated, for trivalent cations (e.g., REEs), organic complexation is better fitted if only the bidentate-binding sites are involved, excluding the monodentate complexation constant (log K_Mm_). The acid–base parameters for HA, the log K_Mb_, and the ΔLK_2_ values are detailed in Table S1. The acid–base parameters were set as generic model values from the “typicalha.mpf” database.^45^ The log K_Mb_ and the ΔLK_2_ were set according to Pourret et al.^10^ and Marsac et al.,^29^ respectively. The ratio of active dissolved organic matter (DOM) to dissolved organic carbon (DOC) was set to 1.65 as the SHM default value.

### 2.3 Adsorption Experiments

Sorption equilibrium experiments were performed to quantify the mass of La, Gd, and Er that adsorb to sand under equilibrium conditions. For each experiment, 1 L of the solution was mixed for 48 h and placed in a flask containing 100 g of sand. All the experiments were conducted in duplicates. The solutions were allowed to mix with sand on a rotating table and sampled daily for 7 days. Control samples were taken before mixing the solution with the sand to determine the initial concentrations of the components of the respective solutions. Solution sampling was conducted for several seconds after manually shaking the experiment bottles, thus allowing the sand to settle while the HA–REE colloids remained suspended in the solution; this enabled the assumption that only REE adsorption is measured, and settled colloids can be dismissed. Thus, decreased REE concentrations in the dissolved phase can be attributed to enhanced REE adsorption on the sand. The sampled solutions were measured for REE concentrations using inductively coupled plasma mass spectrometry (ICP-MS). Adsorbed HA concentrations on the sand were analyzed by drying the sand from each experiment in an oven at 40 °C followed by mixing 2 g of the dry sand with 5 mL of 0.1 M NaOH for 72 h. The HA concentration was analyzed using ultraviolet absorption spectroscopy at λ = 285 nm (UV-1600; Shimadzu Corp.). Additional information on HA calibration is shown in Table S4, Supporting Information. Adsorbed HA results are presented as adsorbed HA mass (mg) on the total mass of sand in the experimental bottles. A mass balance for HA leaching was calculated using the leached HA concentrations and the HA residuals in the experimental solutions. The mass balance was completed with HA recoveries of 92–104%.

### 2.4 Column Transport Experiments

A set of vertical column experiments was conducted to study the mobility and retention of three representative REEs (La, Gd, and Er) under aerobic saturated flow under different physiochemical conditions. Two polycarbonate columns, 19 cm in length and 3 cm in inner diameter, were packed with acid-washed quartz sand and placed vertically. The flow in the columns was from bottom to top, via a multichannel peristaltic pump, at a fixed flow rate of 1 ± 0.05 mL min^–1^. The columns underwent saturation and pH adjustment phases by flowing pH-adjusted DDW through the columns for 48 h prior to the experiment at a flow rate of 0.4 mL min^–1^. As the column completed pH adjustment and the outlet solution pH reached the inlet solution pH value (±0.2 pH value), the DDW was replaced with the experimental solution and effluent collection at the column outlet began.

The experimental solution contained 1 mg L^–1^ of La, Gd, Er, and Br tracers, each, together with various HA concentrations (0, 5, 20, and 50 mg L^–1^). After running the experimental solution for six pore volumes (PVs), the solution was switched back to DDW for two additional PVs. Each experiment was carried out in duplicates. The solution pH of every column throughout the experiment stages was monitored by electrodes placed in the column inlet and outlet. Nitrogen gas was introduced into the inlet solution headspace to minimize exposure to atmospheric CO_2_. The collected samples were measured for La, Gd, Er, and Br concentrations via ICP-MS analysis. The phase in which the solution passes through the column is bounded between the ascent and descent of the Br tracer concentrations. Sand samples from the inlet of the columns were analyzed using scanning electron microscopy (SEM) and energy dispersive X-ray spectroscopy(EDS) analysis to identify the retention of REEs and HA.

### 2.5 REE Vertical Distribution

To examine the vertical distribution of REEs retained in the column, a retention profile was determined by collecting 2 cm sand segments from the column directly at the conclusion of an experiment. The sand was dried at ambient temperature for 4 weeks, then 5 g of dry sand was separated from each segment and placed in 50 mL tubes with 10 mL of 5% nitric acid. The tubes were placed on a rotating table for 72 h, then 3 mL of the solution was sampled for REE concentrations via ICP-MS.

### 2.6 Chemical Analyses

ICP-MS and SEM–EDS were applied for the analysis and are further described in Sections S1 and S2 of the Supporting Information.

### 2.7 Modeling

The BTCs of the various experiments were modeled using Hydrus-1D, a computer software package used widely to model water flow and transport. The program numerically solves the Richards equation for variably saturated water, and the Fickian-based advection–dispersion equation (ADE) for solute transport in porous media. Here, a two-site kinetic model with attachment/detachment mechanisms was applied. Each site has its own attachment and detachment rates, a different retention mechanism, and a different maximum sorbed concentration. This model was chosen due to its use in colloidal transport modeling, which characterized HA transport.^47^ The one-dimensional ADE (eqs S1–S3, Supporting Information) was modified for this two-site kinetic model. An inverse solution of the two-site kinetic model was used to describe the mass transfer of REE between the aqueous and solid phases. The first site (site 1, eq S4, Supporting Information) assumes irreversible time-dependent retention, whereas the second site (site 2, eq S5, Supporting Information) assumes spontaneous reversible retention. For this model, three parameters were fitted: the attachment coefficients of sites 1 and 2 (*k*_a1_ and *k*_a2_, respectively) and the detachment coefficient of site 2 (*k*_d2_). To allow better comparison between the different attachment and detachment mechanisms, *s*_max1_, the maximum sorbed concentration parameter in the time-dependent site (site 1, eq S4, Supporting Information), was fixed to a value of 1.2 μg g^–1^, with *R*^2^ values >0.87 for the fitted curves (Table S3), Supporting Information. The full description of the model is presented in Section S3, Supporting Information.

## 3 Results

### 3.1 REE Speciation

**Figure 1:**
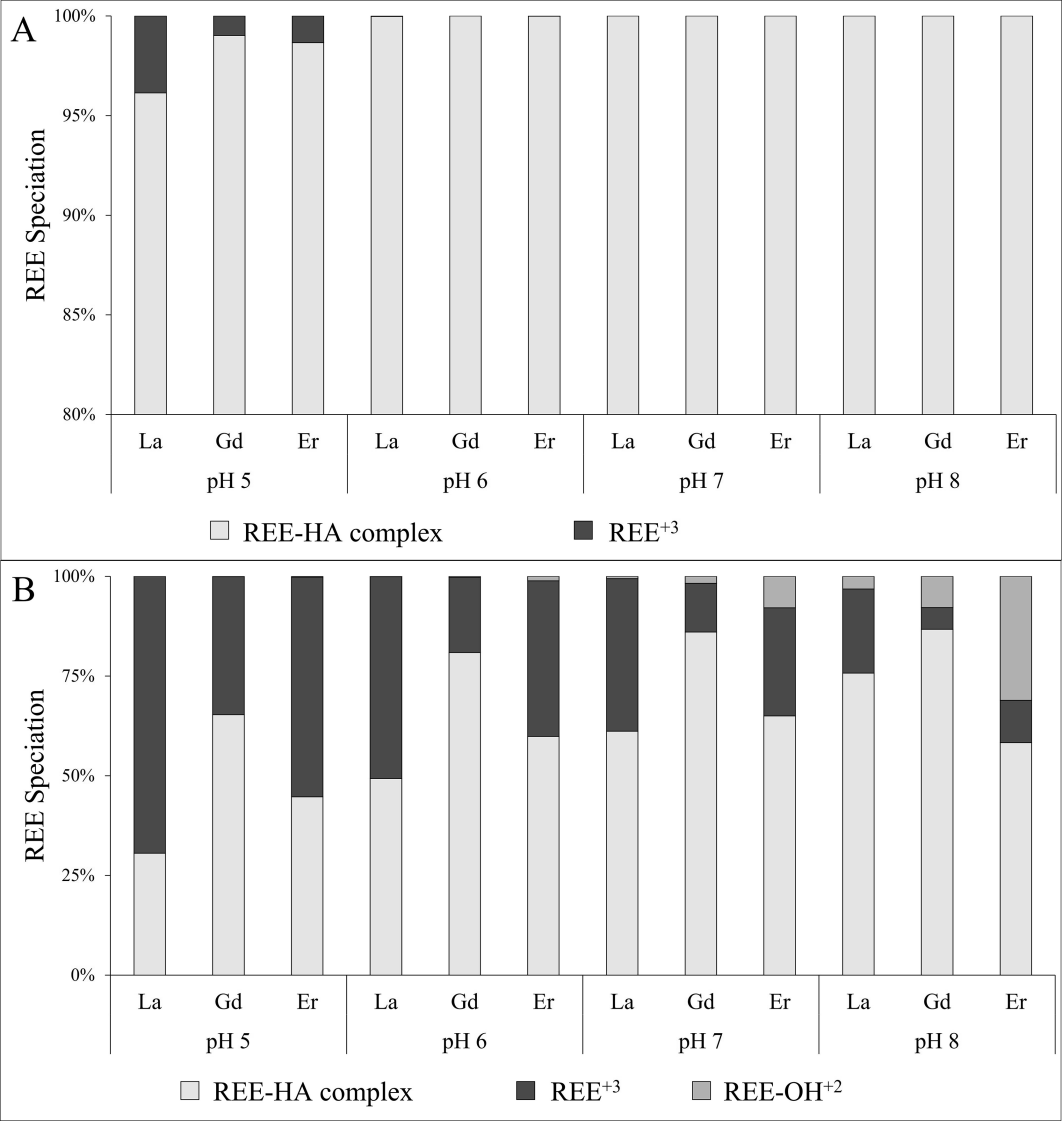
Speciation of La, Gd, and Er in solutions containing (A) 20 mg L^–1^ and (B) 5 mg L^–1^ HA at pH 5–8. La, Gd, and Er concentration is 1 mg L^–1^ for each REE. Complete REE–HA complexation was calculated for 50 mg L^–1^ HA at pH 5–8. The calculations were done using the SHM integrated in Visual MINTEQ version 3.1.

REE speciation calculations with HA in the different experimental solutions were performed using the SHM, integrated into the chemical equilibrium model Visual MINTEQ.^44^ The speciation of La, Gd, and Er in the presence of different HA concentrations 5 and 20 mg L^–1^, at pH 5–8, is presented in Figure 1; REE speciation with 50 mg L^–1^ HA is not shown in Figure 1 because 100% complexation was calculated. The constants used for this modeling are detailed in Table S1, Supporting Information.

The REE speciation analysis reveals that for the 50 mg L^–1^ HA solution, 100% of La, Gd, and Er form complexes with HA at all measured pHs. Complete REE–HA complexation was also observed at pH 6–8 for the 20 mg L^–1^ HA solutions. For the 20 mg L^–1^ HA solution at pH 5, REE complexation with HA was the dominant form with 96.1, 99.0, and 98.6% for La, Gd, and Er, respectively, while the uncomplexed REEs are present as trivalent ions. For the 5 mg L^–1^ HA solutions, the REE–HA fraction decreases as the pH decreases from 7 to 5. In addition, the molar fraction of REE–HA complexes in these solutions is in the order Gd > Er > La, similar to the trend for the 20 mg L^–1^ HA solution at pH 5. For the 5 mg L^–1^ HA solution at pH 8, the Gd–HA fraction is the dominant fraction, followed by La and Er (Gd > La > Er).

The molar fraction of Gd–HA for the 5 mg L^–1^ HA solution is similar at pH 7 and 8, while the molar fraction of La–HA complexes is more significant at pH 8, and the molar fraction of Er–HA complexes is more significant at pH 7. The uncomplexed REE fractions for the 5 mg L^–1^ HA solutions are composed mainly of trivalent REEs at pH 5 and 6 solutions and of a mixture of trivalent REEs and REE–OH^2+^ at pH 7 and 8 solutions.

### 3.2 Adsorption Experiment

**Figure 2:**
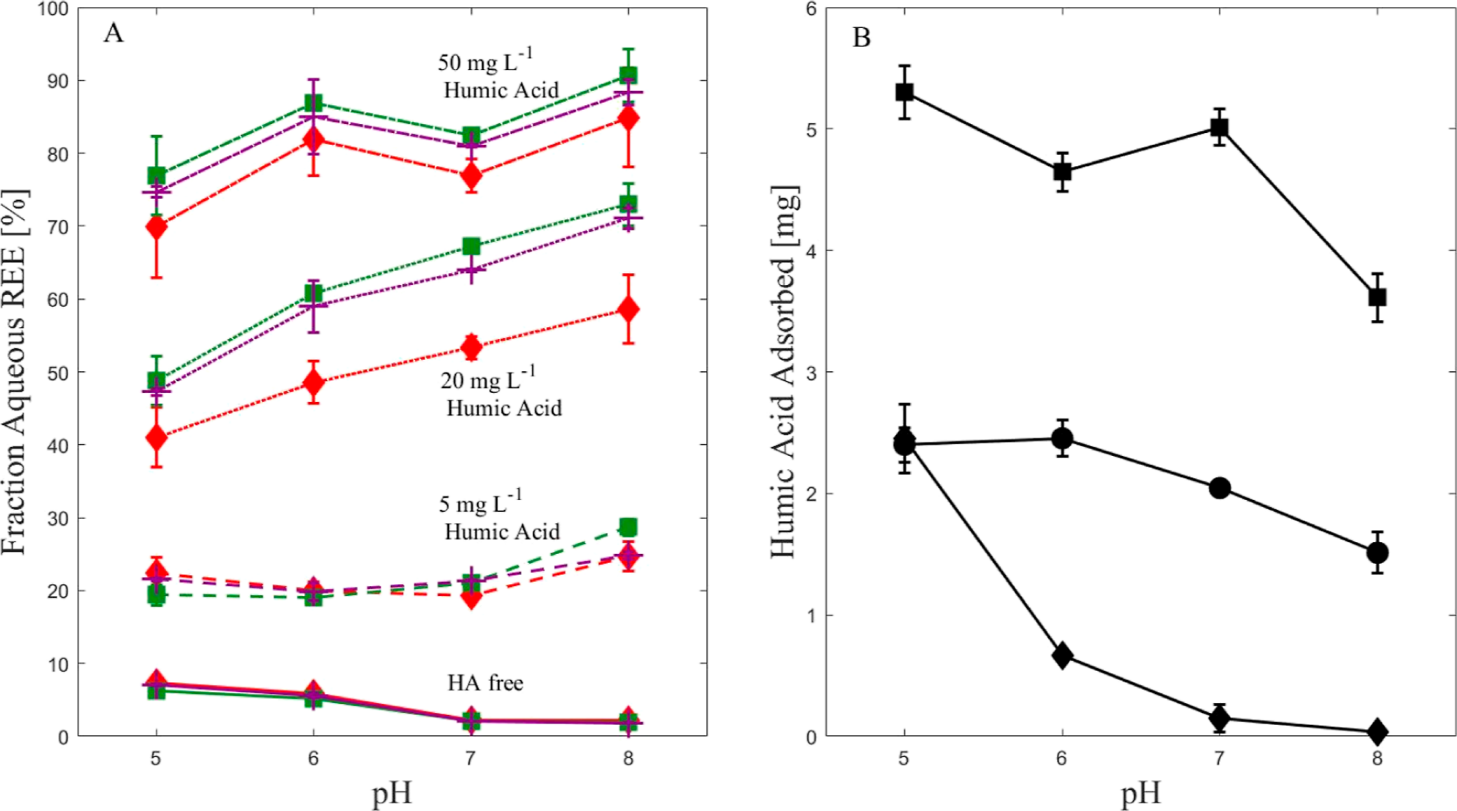
Fraction of dissolved REEs and adsorbed HA mass after 7 days of a batch adsorption experiment. REE concentrations were 1 mg L^–1^ for each REE. HA concentrations were 0, 5, 20, and 50 mg L^–1^. The experiment was conducted at pH 5–8. (A) Fraction of dissolved REEs. Red diamonds: La, green squares: Gd, and purple cross: Er; (B) adsorbed HA mass. Diamonds: 5 mg L^–1^ HA, circles: 20 mg L^–1^ HA, and squares: 50 mg L^–1^ HA. The error bars represent one standard deviation of two measured replicates.

Adsorption dynamics of La, Gd, and Er, for 0, 5, 20, and 50 mg L^–1^ HA, at pH 5–8, are shown in Figures S1–S3, Supporting Information. The comparison between the REE dissolved concentrations and adsorbed HA mass after 7 days of a batch adsorption experiment is shown in Figure 2A,B, respectively. The dissolved REE concentrations and the adsorbed HA mass change as a function of both the HA concentrations and the pH of the solution. In the HA-free solutions, REE concentrations in the dissolved fraction start low (7%) and decrease (to 2%) as the pH increases (Figure 2A). For the HA-containing solutions, REE concentrations in the dissolved fraction increase with HA concentration. The dissolved REE fraction generally increases with pH. The adsorbed HA mass (Figure 2B) increases as more HA is added to the solutions, similar to the dissolved REEs. For the 50 mg L^–1^ HA solutions, the adsorbed HA mass decreases as the solution pH increases from 5 to 6, and then it increases at pH 7, and then decreases again to a minimum value at pH 8. For the 20 mg L^–1^ HA solutions, the adsorbed HA mass starts to decrease at pH 6. For the 5 mg L^–1^ HA solutions, the adsorbed HA mass decreases as the solution pH increases, as the significant drop occurs between pH 5 and 6.

### 3.3 Column Transport Experiments and REE Vertical Distribution

**Figure 3:**
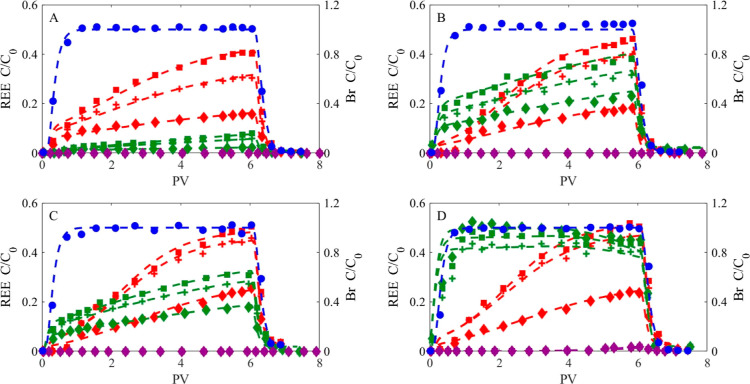
BCT measurements (symbols) and the fitted model (dashed lines) of REEs and the Br tracer under different pH values (5–8) and HA concentrations (5, 20, and 50 mg L^–1^). (A) pH 5, (B) pH 6, (C) pH 7, and (D) pH 8. Red symbols: 50 mg L^–1^ HA concentration. Green symbols: 20 mg L^–1^ HA concentration. Purple symbols: 5 mg L^–1^ HA concentration. La: diamonds. Gd: squares. Er: crosses. Br tracer: circles.

BTCs of La, Gd, Er, and bromide (a conservative tracer) in saturated sand columns and fitted model curves of the BTCs (modeling results presented in Section 3.4) are shown in Figure 3. Column experiment duplicates are shown in Figure S5, Supporting Information.

Complete REE retention for the HA-free solutions was observed in the REE column experiments, for the entire measured pH range (data not shown) and for the 5 mg L^–1^ HA solutions at pH 5–7 (Figure 3A–C, purple symbols). The bromide displayed classical tracer behavior, with a plateau at *C*/*C*_o_ = 1.00 ± 0.05. When REEs did elute from the column (for the experiment with 5 mg L^–1^ HA solutions: pH 8; 20 and 50 mg L^–1^ HA solutions: pH 5–8), a clear difference in the REE BTCs was found as the key components of the inlet solution chemistry were changed (pH and HA concentrations). In most cases, Gd mobility was the highest, followed by Er and La.

For the 50 mg L^–1^ HA solutions (Figure 3A–D, red symbols; and Table S2), REE fractions in the effluent increased with the duration of the experiment for all measured pHs. The mobility of the different REEs increased with the solution pH from 5 to 8. The slope of the REE BTCs becomes steeper, and the Er BTC pattern approaches that of the Gd. Furthermore, a 0.7 PV delay in REE elution was observed at pH 6–8, compared to no delay in the pH 5 solution.

For the 20 mg L^–1^ HA solutions, REE mobility varies among the different pHs in the following order (pH) 8 > 6 > 7 > 5 (Figure 3A–D, green symbols; Table S2). The BTC shape changed for the 20 mg L^–1^ HA solutions. At pHs 5, 6, and 7, REE eluted concentrations increase throughout the duration of the experiment, similar to the REE column experiment with 50 mg L^–1^ HA. On the other hand, for the 20 mg L^–1^ HA solutions at pH 8, REEs reached maximum concentration values after 0.7 PV, followed by a moderate decrease in eluted REE concentrations. For the 5 mg L^–1^ HA solutions, REE mobility at pH 8 was low (0.54 ± 0.03%, Table S2), and a 4.5 PV delay in REE elution was observed (Figure 3D, purple symbols).

The vertical REE distribution profiles of the different column experiments (Figure S4, Supporting Information) show variations in the adsorption patterns of the REEs. In general, REEs remain near the inlet. More acidic conditions result in a longer spread of the REEs in the column, reaching distances of 7, 5, 3, and 1 cm from the inlet for pH 5, 6, 7, and 8, respectively. In addition, at pH 6–8 (Figure S4B–D, Supporting Information), the highest adsorption was measured for the 5 mg L^–1^ HA concentrations, followed by the 20 mg L^–1^ HA solutions, while minimal adsorption occurs for the 50 mg L^–1^ HA solutions. At pH 5 (Figure S4A, Supporting Information), REE retention is similar for 20 and 5 mg L^–1^ HA solutions, and REE adsorption for the 50 mg L^–1^ HA solution occurred in the top 3 cm.

**Figure 4:**
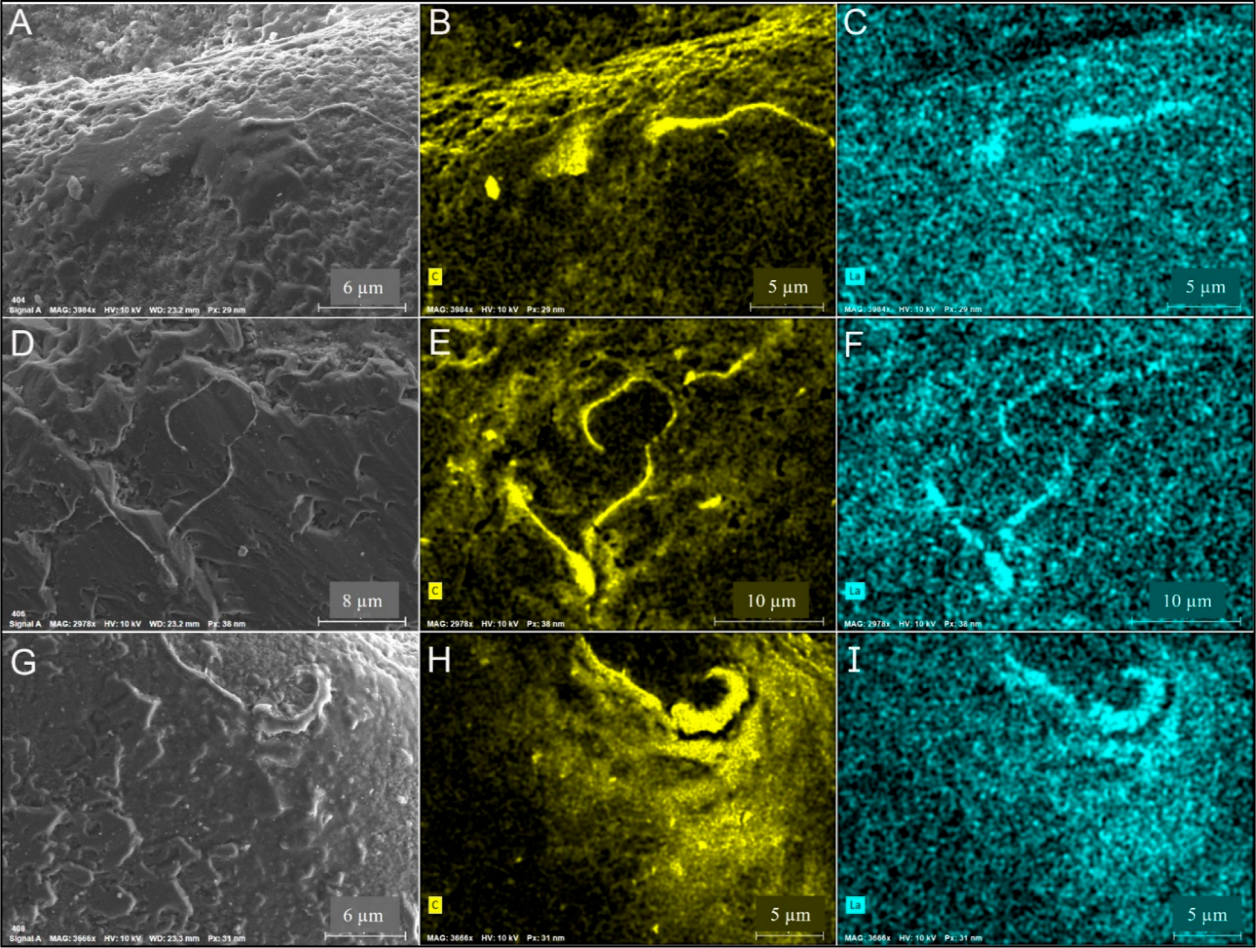
SEM images (A,D,and G) and their correlated X-ray intensity maps of carbon (B,E,H) and lanthanum (C,F,I), collected from three different particles (#1: A–C; #2: D–F; and #3:G–I). Elongated regions with high carbon (HA) and La concentrations are considered REE–HA complexes attached to the sand.

The elemental analysis and electron imaging were conducted using SEM and EDS for sand samples obtained from the inlet of the columns. Figure 4 shows three surfaces of sand particles within the sample, together with X-ray intensity maps of carbon (indicating the presence of HA) and La (as a representative REE). The elemental analysis of each surface shows regions with enhanced amounts of La correlated to regions with enhanced amounts of C, appearing mostly in elongated regions of length 5–20 μm attached to some of the elongated ridges present on the sand surface (Figure 4A,D,G). In this context, it is noted, too, that no correlation was observed between La and other detected elements such as Fe, K, and P.

### 3.4 BTC Modeling Results

The BTC model fits of the column experiments are shown in Figure 3. For this model, three parameters were fitted: the attachment coefficients of sites 1 and 2 (*k*_a1_ and *k*_a2_, respectively) and the detachment coefficient of site 2 (*k*_a2_). Figure S6, Supporting Information, shows the fitted coefficients for Gd, as a representative REE, while the values of the fitted coefficients for La, Gd, and Er are detailed in Table S3, Supporting Information. The fitted coefficients for Gd BTCs in all the experiments in which REEs were eluted from the columns (Figures 3 and S6, Supporting Information) show that for the 50 mg L^–1^ HA solutions at all pHs, *k*_a1_ values are higher than *k*_d2_ values. In these solutions, *k*_a2_ values increase slightly as the solution pH decreases, while the *k*_d2_ values show the opposite trend. *k*_a1_ values increase as the solution pH drops from pH 8 to pH 6, then drops to a minimum value at pH 5. For the 20 mg L^–1^ HA solutions, *k*_a1_ and *k*_a2_ fitted values are similar and increase as the solution pH decreases, with higher *k*_a2_ at pH 7. For these solutions, *k*_a2_ values show the opposite, decreasing values with decreasing pH, besides higher *k*_a2_ at pH 7. For the 5 mg L^–1^ HA solution at pH 8, *k*_a1_ values are 1 order of magnitude higher than *k*_a2_ values.

## 4 Discussion

### 4.1 REE Speciation

Calculation of REE speciation using SHM (Figure 1) indicates complete or near-complete (>95%) complexation of REEs with HA at concentrations of 20 and 50 mg L^−1^ HA. For the 5 mg L^−1^ solutions, the amount of REE-HA complexes decreases as the acidity of the solution increases. The complexation of REEs with HA was studied previously by Pourret et al.,^21^ who examined REE–HA complexation at pH 2.18–10.44 and for HA concentrations of 5, 10, and 20 mg L^–1^. These authors reported complete complexation of REEs with HA for 20 mg L^–1^ HA concentrations at pH > 4, and a gradual increase in the amount of REE–HA complexes as a function of pH for 5 mg L^–1^ HA concentrations. The speciation analysis using the SHM in this study (Figure 1) agrees with Pourret et al.,^21^ presenting similar complexation results. REE complexation for 50 mg L^–1^ HA concentrations was not studied by Pourret et al.^21^ However, the increase in the amount of REE–HA complexes with increasing HA concentrations, accompanied by the complete complexation for 20 mg L^–1^ HA observed by Pourret et al.,^21^ supports the SHM calculations of complete REE complexation with HA for a system with 50 mg L^–1^ HA in the solution. Pourret et al.^21^ concluded that the amount of REE–HA complexes increases with HA concentration and solution pH due to the deprotonation of the HA carboxylic and phenolic functional groups.

The formation of the first REE hydrolysis product, REE–OH^2+^, which is more dominant at higher pH, causes a change in the REE–HA complexation pattern. The first REE hydrolysis constant increases with the lanthanide atomic number, resulting in a higher amount of Er–OH^2+^ than La–OH^2+^ for a specific pH. At pH < 8, where the amount of REE–OH^2+^ was relatively small (<7%), the REE–HA complexation pattern was Gd > Er > La. At pH 8, the amount of Er–OH^2+^ increased from 8 to 31%, while the amount of La–OH^2+^ increased from 0.6 to 3.2%, resulting in the change of the REE–HA pattern to Gd > La > Er.

The speciation calculation here indicates that when only some of the REEs in the solution are complexed to HA, each tested element produces a unique collection of species. The fraction of Gd complexes with HA was the largest, followed by Er and La. The higher complexation of MREE (e.g., Gd) compared to Er and La was reported previously^10,16,21,29,48,49^ in REE–HA complexation experiments with a high REE/HA ratio, similar to the observation here.

### 4.2 Inorganic REE Retention

High retention of REEs in the absence of HA in the solution was observed in both the batch adsorption and column transport experiments. In the column transport experiments, complete REE retention (no analyte elution from the column; data not shown) was found for a pH range of 5–8. High inorganic REE retention on sand was reported previously.^31,50−52^ Tang and Johannesson^52^ reported increased REE adsorption as a function of solution pH, with almost 100% of the REEs being adsorbed at pH 5.5. REE adsorption increases with pH due to mineral surface deprotonation, promoting positively charged REE adsorption by ion exchange or surface complexation.^24^ Kim et al.,^50^ Randall et al.,^51^ and Yoshida and Suzuki^31^ used Eu as a representative REE in sand column experiments and reported complete inorganic Eu retention. This current study shows that REEs are not mobile in saturated sand columns without the addition of HA to the solutions at slightly acidic to slightly alkaline solutions.

### 4.3 HA Concentrations and Solution pH Effect on REE Retention and Mobility

#### 4.3.1 REE Adsorption on Sand

The adsorption experiment results show that the dissolved REE concentrations increase as more HA is added to the solution (Figure 2A), indicating that HA addition inhibits REE adsorption on the sand. In this experiment, the adsorbed HA mass (Figure 2B) increases as the HA concentrations increase. This observation suggests that the mechanisms controlling REE adsorption in the presence of HA could be (1) electrostatic repulsion between the HA in the solution and the adsorbed HA, which hinders additional adsorption of REE–HA complexes, (2) reduction in the number of sorption sites on the sand due to higher adsorbed HA, and/or (3) stronger binding of REEs to the HA than the sand, which results in less adsorption. In addition, Figure 2B shows that the adsorbed HA mass generally decreases with increasing solution pH for each specific HA concentration, although different trends were observed for the various HA concentrations. This link between adsorbed HA and REEs indicates that REE–HA complexes are co-adsorbed on the sand, as suggested previously.^53^ The observed trend for HA adsorption as a function of pH can be explained by the deprotonation of HA at elevated pH. HA molecules are protonated at pH < 4, with an aggregated structure. At pH values between 4 and 6, the humic structure is more extended and dispersed as the charge is close to zero. At pH > 6, HA molecules are deprotonated, yielding a negative charge density on the molecule. The continuous deprotonation of HA with increasing pH decreases HA adsorption on the sand as the solution pH increases.^54,55^

The REE–HA adsorption on quartz is controlled mainly by the proportions of REE–HA complexes and the adsorption of HA on quartz surfaces, both of which are binary systems affected by the solution pH and HA concentrations. The complexation of REE with HA was discussed in detail above. The adsorption of HA on quartz sand at different pH values was studied by Fairhurst et al.^56^ and Lippold et al.,^24^ who reported that HA adsorption on quartz sand decreases with a rise in solution pH for pH values above 5. As the pH increases, both the sand and the HA functional groups become increasingly negatively charged, and electrostatic repulsion increases. This repulsion is also valid for slightly acidic pH, as HA colloids were shown to be negatively charged across the tested pH range (pH 5–8), and the quartz isoelectric point is around pH 3.^56,57^ For this reason, electrostatic interaction could not be the sole mechanism that controls HA adsorption on sand, as HA adsorption does occur throughout the experimental pH range in this study (pH 5–8; Figure 2B). Other mechanisms reported to be involved in HA adsorption include van der Waals attraction,^58,59^ hydrophobicity at high pH,^60,61^ hydrogen bonding,^62,63^ and ligand exchange or surface complexation between acidic groups of the quartz sand and HA.^64,65^

In the ternary HA–REE–quartz systems, the elevated solution pH increases the electrostatic barrier for interactions between the HA colloids and the quartz. The elevated pH also enhanced the dissociation of the HA functional groups. These two parallel processes lead to a gain in metal-binding sites on the HA colloids in the solution. Consequently, a competitive situation is established between metal adsorption and the formation of REE–HA complexes, and REE adsorption decreases. Lowering the solution pH would cause enhanced adsorption of REEs on quartz, as more HA would be adsorbed and mediate the REE co-adsorption on the quartz sand surface.^24,66,67^

Retained REEs on the sand grains were detected using SEM and EDS analysis (Figure 4). These retained REEs were observed in locations in which relatively high concentrations of carbon were also detected. These regions are considered REE–HA complexes, indicating that REE retention on sand is caused by the co-adsorption of REE and HA in the form of REE–HA complexes. The co-adsorption of REE–HA complexes, which was observed using SEM and EDS, is supported by the similar behavior (co-adsorption of REE–HA complexes) in the batch adsorption experiment (Section 3.2).

#### 4.3.2 REE Transport through Sand Columns

The column transport experiment results (Figure 3) generally show that REE mobility increased with increasing solution pH and with the addition of HA to the solutions, which corresponds to the adsorption patterns of REEs on quartz sand in the presence of HA (Figure 2). The mobility of REEs in sand columns in the presence of humic materials was studied previously.^16,50,51^ Randall et al.^51^ reported that only Eu–HA complexes were eluted from the column, while trivalent Eu was retained entirely. Pédrot et al.^16^ showed that humic substances, mainly HA, control REE mobility through sand columns.

REE mobility in the presence of 50 mg L^–1^ HA increases as the solution pH increases, presenting a similar BTC shape through all measured pHs. In contrast, the 20 mg L^–1^ HA solution BTCs show different mobility patterns for the various pHs. At slightly acidic to neutral pH, the REE BTCs are similar to those observed at higher HA concentrations (Gd > Er > La), and the REE mobility is in the order of pH 6 > 7 > 5. At pH 8, the BTC shape changed, as the maximum concentration values were measured after 0.7 PV, and not at the column wash step as described previously. Although REE mobility drops at pH 7, it increases at pH 8, exceeding REE mobility in the parallel solution with higher HA concentrations. A similar mobility pattern at different pHs (pH 8 > 6 > 7 > 5)—observed in column experiments with the addition of 20 mg L^–1^ HA (Figure 3)—was observed in an adsorption experiment conducted with the addition of 50 mg L^–1^ HA (Figure 2). This indicates the difference in REE–HA retention mechanisms between equilibrium batch adsorption and nonequilibrium column transport experiments. In addition, these differences in REE retention between the adsorption experiment and the column transport experiments, and the change in the BTC shape as the pH increased from 7 to 8, suggest that the REE solution with 20 mg L^–1^ HA is more sensitive to small chemical and physical changes that occur as the solution passes through the sand column. The speciation calculation supports this conclusion for the 20 mg L^–1^ HA solution, for which complete REE–HA complexation was calculated for pH of 6 but not for pH of 5. These differences are not observed in the 50 mg L^–1^ HA solutions, where the BTC shape is similar for all pHs, in agreement with the speciation calculations. For the 5 mg L^–1^ HA experiments, REEs are eluted from the column only at pH 8.

### 4.4 REE Retention Pattern

Mobility and retention experiments conducted in this study show that in the presence of HA, the three tested REEs have different mobilities for a specific HA concentration and solution pH conditions. The observed REE pattern (Gd > Er > La) corresponds to the REE–HA complexation pattern, which was observed for all the solutions in which partial REE–HA complexation was calculated (20 mg L^–1^ pH 5, 5 mg L^–1^ pH 5–8; Figure 1), which shows that Gd forms the largest amount of complexes with HA, followed by Er and La.

A similar REE pattern was reported by Pédrot et al.,^16^ who conducted REE transport experiments in a sand column with organic matter. The most dominant size fraction in Pédrot et al.^16^ experiments was associated with HA. The link between the REE complexation patterns and the REE adsorption patterns in the presence of HA indicates that REE–HA complex formation controls REE retention and mobility in quartz sand. More specifically, the formation of REE–HA complexes hinders REE retention on sand.

A different REE retention pattern was observed in the column experiment for the 20 mg L^–1^ HA solution at pH 8. Lanthanum was the most mobile for these experimental conditions, followed by Gd and Er (Figure 3D). Assuming that REE–HA complexation controls REE mobility, this fractionation pattern implies that the amount of REE–HA complexes decreases from La to Gd and Er, contrary to our calculated complexation using the SHM (Figure 1). Sonke and Salters^8^ reported a similar complexation pattern in the REE–HA complexation experiment conducted at much lower REE/HA ratios. This change in REE patterns between the adsorption experiment (Gd > Er > La) and the column experiments (La > Gd > Er) could be related to the sensitivity of the 20 mg L^–1^ solutions. These solutions are sensitive to even small changes in the solution chemical conditions, evident from the speciation calculation, in which complete complexation was calculated at pH 6 but not at pH 5. As the solution passes through the column, the chemical conditions change slightly, and more hydrolysis products might compete with REE–HA complexation. REE hydrolysis might lead to a change in the amount of REEs that form complexes with HA. The observed speciation calculation (Figure 1) is in accordance with the formation of more hydrolysis products for Er than for La.

### 4.5 BTC Analysis and Modeling

The three major phases of the BTC could be assigned to (i) increasing REE concentrations in the eluted solution during the experiment. This phase could be described as a time-dependent behavior, controlled by the time-dependent attachment coefficient, *k*_a1_ and *s*_max_ the maximum sorbed concentrations; (ii) a constant REE elution, referred to as a “plateau”, that is governed by the attachment coefficient, *k*_a2_; and (iii) the tail of the BTC, following the column wash step. The detachment coefficient *k*_d2_ controls this phase. In the attachment/detachment model, the fitted attachment coefficients, *k*_a1_ and *k*_a2_, control the slope of the BTC, which is a function of the eluted REEs and the experiment duration.

The BTC shape for all the 50 mg L^–1^ HA solutions shows an increase in the eluted REE concentration with time (Figure 3, red lines), yielding higher fitted values of *k*_a1_ than *k*_a2_ (Figure S6, Supporting Information). In these experiments, *k*_a1_ values increase slightly as the pH drops from 8 to 6, while *k*_a2_ values remain similar due to the increased REE adsorption. For the 50 mg L^–1^ HA solution at pH 5, the BTC approaches a “plateau” toward the end of the experiment, which results in lower *k*_a1_ values.

For the 20 mg L^–1^ HA solutions, an increase in the eluted REE concentration with time is observed for pH 5, 6, and 7, where *k*_a1_ values are higher than *k*_a2_ values. On the other hand, at pH 8, REE eluted concentrations increase only at the first PV, followed by a near-plateau behavior. Therefore, *k*_a2_ fitted values are higher than *k*_a1_ values. In addition, higher attachment coefficients are observed at pH 5 and 7 due to the increased REE retention. For the 5 mg L^–1^ HA solution at pH 8, *k*_a2_ fitted values are 1 order of magnitude higher than *k*_a1_ values due to the minimal REE elution and the near-plateau shape of the BTC.

The values of the fitted detachment coefficient of site 2, *k*_d2_ (Figure S6, Supporting Information), are 2–3 orders of magnitude lower than the attachment coefficient values, which indicate that the detachment mechanism is negligible. This can also be observed from the shape of the BTC tail, as no significant amounts of REEs are eluted from the column at the column wash step after six PVs. For the 50 mg L^–1^ HA solutions, *k*_d2_ values decrease with decreasing pH, indicating weaker adsorption at higher pH.

## 5 Conclusions

The retention and mobility of La, Gd, and Er in saturated quartz sand columns were studied under different solution pH and HA concentrations, to provide insights into the mechanisms that control REE mobility and retention in porous media. Here, REEs were shown to be more mobile as the HA concentrations and the solution pH increased. All three tested REEs were immobile without the addition of HA to solutions at pH 5–8. The retention and mobility of REEs were shown to be controlled by the formation of REE–HA complexes, which are more abundant in higher pH and HA concentrations. Complexation calculations conducted using the SHM indicated that Gd exhibits the most significant degree of complexation with HA among the tested REEs, followed by Er and La. This REE–HA complexation pattern (Gd > Er > La), which reflects an organic matter-mediated complexation pattern, leads to weaker Gd retention in porous media compared to Er and La. These differences in REE retention were also observed in solutions where complete REE–HA complexation was calculated, indicating the strong effect of REE–HA complexes on REE retention and mobility. The correlation found between adsorbed HA and REEs, observed in batch adsorption experiments, indicates that REE–HA complexes co-adsorb on the quartz sand. REEs were found to be retained on the sand in locations where relatively high carbon concentrations were detected (indicating the presence of HA), indicating the co-absorbance of REE–HA complexes. The modeling of the BTCs of La, Gd, and Er suggests that the attachment mechanisms involved in REE retention are both time dependent and spontaneous, as the time-dependent mechanism is more dominant in higher HA concentrations. These observations imply that at slightly acidic to slightly alkaline pH, REEs may be mobile in groundwater systems with high HA concentrations. In contrast, in HA-poor systems, REEs are expected to be retained.

## Supporting Information Available

Additional experimental results and model description (PDF)

## Abbreviations

REE: rare earth elements
PV: pore volume
BTC: breakthrough curve
HA: humic acid
SHM: Stockholm Humic Model
DOM: dissolved organic matter
DOC: dissolved organic carbon
