# Supplementary material for: Mobility and Retention of Rare Earth Elements in Porous Media
Source: ACS Omega. 2022 Jun 2;7(23):19491–501. doi: 10.1021/acsomega.2c01180 (PMC9202298; doi:10.1021/acsomega.2c01180)
Supplement: Supplementary file 1 — ao2c01180_si_001.pdf [file ao2c01180_si_001.pdf]

Supplementary Materials

Mobility and retention of rare earth elements in porous media

Nitai Amiel*, Ishai Dror, and Brian Berkowitz

Department of Earth and Planetary Sciences, Weizmann Institute of Science, Rehovot 7610001, Israel

The Supplementary Material contains information on chemical analyses, followed by model description of breakthrough curve (BTC) modeling using Hydrus 1-D. We present Figures and tables showing adsorption dynamics of La, Gd, and Er in adsorption batch experiments (Figure S1-S3), retention profiles of the different column experiments (Figure S4), and duplicates of the column experiments (Figure S5). The fitted model parameters for Gd in Figure S6. The list of constants used for REE speciation using the Stockholm Humic Model, the list of conditions applied, mass recovery for the entire set of REE column experiments, the attachment and detachment coefficients of column experiment BTC modeling for La, Gd, and Er, and the Humic acid calibration data are given in Tables S1, S2, S3, and S4, respectively.

S1. ICP-MS analysis

All samples were analyzed via ICP-MS (Agilent 7700s) for lanthanum, gadolinium, erbium, and bromide concentrations. Drift corrections were carried out using europium as an internal standard and by repeatedly analyzing a calibration solution of 500 μg L^{-1} concentration as a drift monitor throughout the analysis. Memory effects were avoided by additional manual cleaning using 5% HNO_3. To eliminate mass interferences for Gd and Er, the following masses were measured: Gd^{155}, Gd^{157}, and Gd^{160}; and Er^{166}, Er^{168}, and Er^{170}.

S2. Scanning electron microscopy (SEM) and elemental mapping

Elemental analysis of the samples was performed using energy dispersive x-ray spectroscopy (EDS). The EDS (Bruker FlatQUAD 4 quadrants SDD detector) is installed on a Zeiss Sigma500 scanning electron microscope (SEM). The EDS data were collected with an accelerating voltage of

10kV using the mapping application, where both SEM image and x-ray spectra were acquired simultaneously. The x-ray map of each element can be color-coded and presented as an intensity map or heat map.

S3. Modeling

S3.1 Model description

The breakthrough curves (BTCs) of the various experiments were modeled using Hydrus-1D, a computer software package used widely to model water flow and transport. The program numerically solves the Richards equation for variably saturated water and the Fickian-based advection-dispersion equation for solute transport in porous media.

Here, a two-site kinetic model with attachment/detachment mechanisms was applied. Each site has its own attachment and detachment rates, a different retention mechanism, and a different maximum sorbed concentration. This model was chosen due to its use in colloidal transport modeling, which characterizes HA transport [1].

The one-dimensional advection-dispersion equation (ADE) was modified for this two-site kinetic model; the mass balance equation is defined as (Šimůnek and van Genuchten, 2008):

$$\frac{\partial(\theta C)}{\partial t} + \rho\frac{\partial S1}{\partial t} + \rho\frac{\partial S2}{\partial t} = \frac{\partial}{\partial z}\left(\theta D\frac{\partial C}{\partial z}\right) - \frac{\partial(qC)}{\partial z} - \phi \qquad (1)$$

where θ is water content [mL(water) mL(soil)$^{-1}$], C is the concentration in the liquid phase [μg mL^{-1}], t is time [min], ρ is soil density [g cm^{-3}], s is the sorbed concentration on the sorption sites [μg g^{-1}], z is the distance from column inlet [cm], D is the hydrodynamic dispersion coefficient [cm^2 min^{-1}], q is the fluid flux [cm min^{-1}], and ϕ is the inactivation processes in the liquid phase [μg (mL min)$^{-1}$]. The sorbed concentrations ($S1$ and $S2$) are functions of attachment and detachment coefficients (k_a and k_d, respectively; Equation 2) and a time-dependent retention function, ψ, which is based on the Langmuir equation (Equation 3), where s_{max} is the maximum solid-phase concentration:

$$\rho \frac{\partial s}{\partial t} = k_a \psi \theta c - k_d \rho S \quad (2)$$

$$\psi = \left(1 - \frac{s}{s_{max}}\right) \quad (3)$$

An inverse solution of the two-site kinetic model was used to describe the mass transfer of REE between the aqueous and solid phases. The first site (site 1, Equation (4)) assumes irreversible time-dependent retention, whereas the second site (site 2, Equation (5)) assumes spontaneous reversible retention as:

$$\rho \frac{\partial s1}{\partial t} = k_{a1} \psi \theta c \; ; \; \psi = \left(1 - \frac{s}{s_{max1}}\right) \quad (4)$$

$$\rho \frac{\partial s2}{\partial t} = k_{a2} \theta c - k_{d2} \rho S \quad (5)$$

The hydrodynamic dispersion coefficient, D=3 [cm^2 min^{-1}], was calculated by fitting the bromide tracer results to the classic advection-dispersion model solution. First, the data were fitted to four retention parameters (k_{a1}, s_{max1}, k_{a2}, k_{d2}), with $R^2 > 0.95$ in all cases. Then, the s_{max1} value was fixed to an average value to reduce the number of fitted parameters values, if applicable ($R^2 > 0.87$).

Tables

Table S1: List of constants used for REE speciation using Stockholm Humic Model.

Parameter	Description	Values
n_A	Amount of type-A sites (mol g^{-1})	3.55E-03
n_B	Amount of type-B sites (mol g^{-1})	1.78E-03
log K_A	Intrinsic proton dissociation constant for type-A sites	-4.13
log K_B	Intrinsic proton dissociation constant for type-B sites	-8.99
ΔpK_A	Distribution term that modifies log K_A	3.03
ΔpK_B	Distribution term that modifies log K_B	3.03
log K_{Mb}	Intrinsic equilibrium constant for bidentate complexation of metal M	La: -5.66; Gd: -5.04; Er: -5.65
ΔLK_2	Distribution term that modifies the strength of complexation sites	La: 1.1; Gd: 1.35; Er: 1.7
r	Molecular radius	1.8 nm
C	Stern layer capacitance	2 F m^{-2}
N_s	Site density of HS functional groups	1.2 sites nm^{-2}
g_f	Gel fraction parameters	0.78
K_C	Intrinsic equilibrium constant for accumulation of screening counterions	$10^{0.8}$

*The monodentate complexation constant (log KMm) was excluded from the model.

Table S2: List of conditions applied and mass recovery for the entire set of REE column experiments. La, Gd, and Er concentrations were 1 mg L^{-1} for each REE.

Experiment #	pH	Humic Acid concentrations [mg L^{-1}]	La	Gd	Er
			Eluted phase recovery [%]	Eluted phase recovery [%]	Eluted phase recovery [%]
1	5	HA-FREE	-	-	-
2	6	HA-FREE	-	-	-
3	7	HA-FREE	-	-	-
4	8	HA-FREE	-	-	-
5	5	5	0	0	0
6	6	5	0	0	0
7	7	5	0	0	0
8	8	5	0.50±0.02	0.48±0.05	0.37±0.03
9	5	20	1.8±0.3	4.8±0.6	3.6±0.3
10	6	20	17.3±0.2	29.3±0.4	23.8±0.3
11	7	20	13.2±0.4	23.4±0.3	20.1±0.5
12	8	20	47.0±3.2	44.4±1.5	40.5±2.4
13	5	50	12.2±2.6	29.7±4.1	22.7±1.8
14	6	50	12.6±3.1	29.2±2.4	25.6±2.2
15	7	50	14.6±3.6	31.5±2.1	29.3±1.3
16	8	50	15±2.6	34.2±1.1	31.4±2.4

Table S3: Attachment and detachment coefficients of column experiments BTC modeling using Hydrus 1-D.

	**50 mg L^{-1} HA**											
	pH 8			pH 7			pH 6			pH 5		
	La	Gd	Er	La	Gd	Er	La	Gd	Er	La	Gd	Er
k_{a1}	2.05E-01	1.89E-01	1.79E-01	2.34E-01	2.12E-01	2.00E-01	1.84E-01	2.19E-01	2.06E-01	1.02E-01	1.13E-01	1.11E-01
k_{a2}	9.94E-02	4.61E-02	5.12E-02	9.97E-02	5.02E-02	5.45E-02	1.10E-01	5.38E-02	6.17E-02	1.18E-01	5.40E-02	7.20E-02
k_{d2}	4.57E-04	4.19E-04	3.37E-04	4.56E-04	3.99E-04	3.21E-04	1.35E-04	2.03E-04	1.64E-04	1.69E-04	1.14E-04	1.51E-04
R^2	0.976	0.990	0.987	0.974	0.989	0.986	0.962	0.987	0.983	0.977	0.992	0.987
	**20 mg L^{-1} HA**											
	pH 8			pH 7			pH 6			pH 5		
	La	Gd	Er	La	Gd	Er	La	Gd	Er	La	Gd	Er
k_{a1}	2.49E-02	2.44E-02	2.45E-02	1.05E-01	8.99E-02	9.36E-02	8.28E-02	6.16E-02	7.00E-02	1.48E-01	2.27E-01	2.02E-01
k_{a2}	2.00E-02	2.58E-02	3.29E-02	1.11E-01	7.02E-02	7.97E-02	8.73E-02	5.13E-02	6.47E-02	3.00E-01	1.80E-01	2.03E-01
k_{d2}	2.01E-04	3.74E-04	4.16E-04	3.40E-04	2.74E-04	2.86E-04	4.71E-04	3.53E-04	3.67E-04	2.71E-04	3.70E-04	3.39E-04
R^2	0.963	0.964	0.964	0.965	0.984	0.981	0.875	0.959	0.937	0.871	0.951	0.939
	**5 mg L^{-1} HA**											
	pH 8			pH 7			pH 6			pH 5		
	La	Gd	Er	La	Gd	Er	La	Gd	Er	La	Gd	Er
k_{a1}	2.51E+00	2.25E+00	2.52E+00	No BTC								
k_{a2}	2.59E-01	2.66E-01	2.70E-01									
k_{d2}	3.03E-04	3.48E-04	3.13E-04									
R^2	0.903	0.899	0.908									

Table S4: Humic acid measurement calibration data

Concentration (mg L^{-1})	Readings
0.01	-0.0141
0.05	-0.0009
0.1	0.0080
0.5	0.0122
1	0.0256
2	0.0417
5	0.1076
10	0.2013
20	0.4209
50	1.1234
100	2.2636

Calibration eqn: Abs = 0.02264*Conc -0.00746

Correlation Coefficient 0.99972

FIGURES

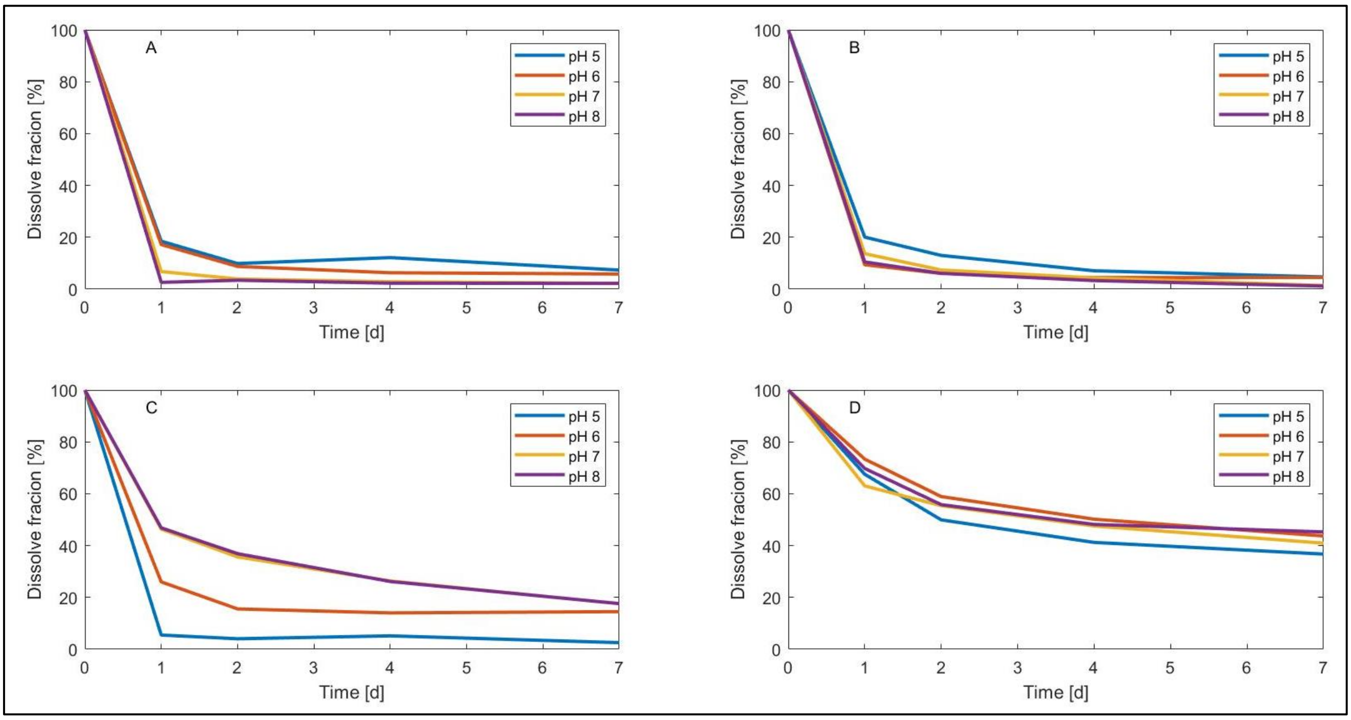

Figure S1. Adsorption dynamics of La in batch experiments under different pH values (5-8) and humic acid (HA) concentrations. (A) 50 mg L^{-1} HA, (B) 20 mg L^{-1} HA, (C) 5 mg L^{-1} HA, (D) HA-free.

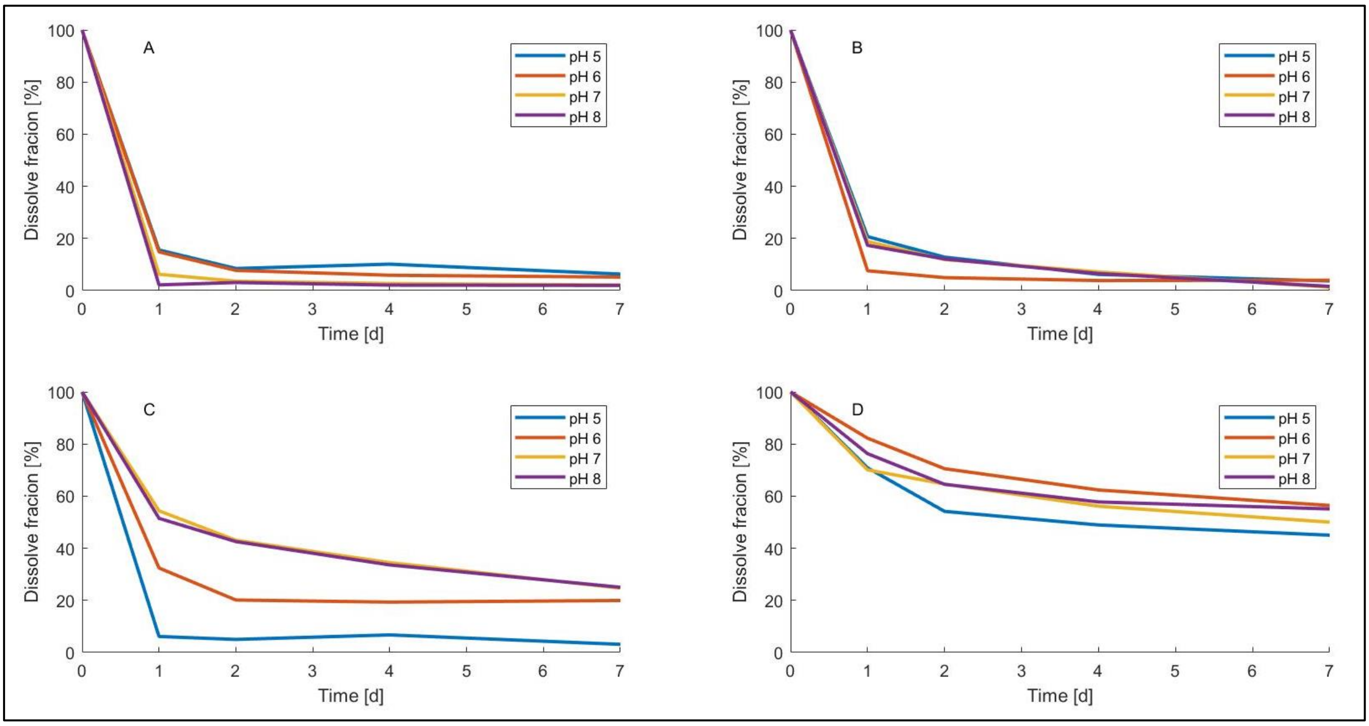

Figure S2. Adsorption dynamics of Gd in batch experiments under different pH values (5-8) and humic acid (HA) concentrations. (A) 50 mg L^{-1} HA, (B) 20 mg L^{-1} HA, (C) 5 mg L^{-1} HA, (D) HA-free.

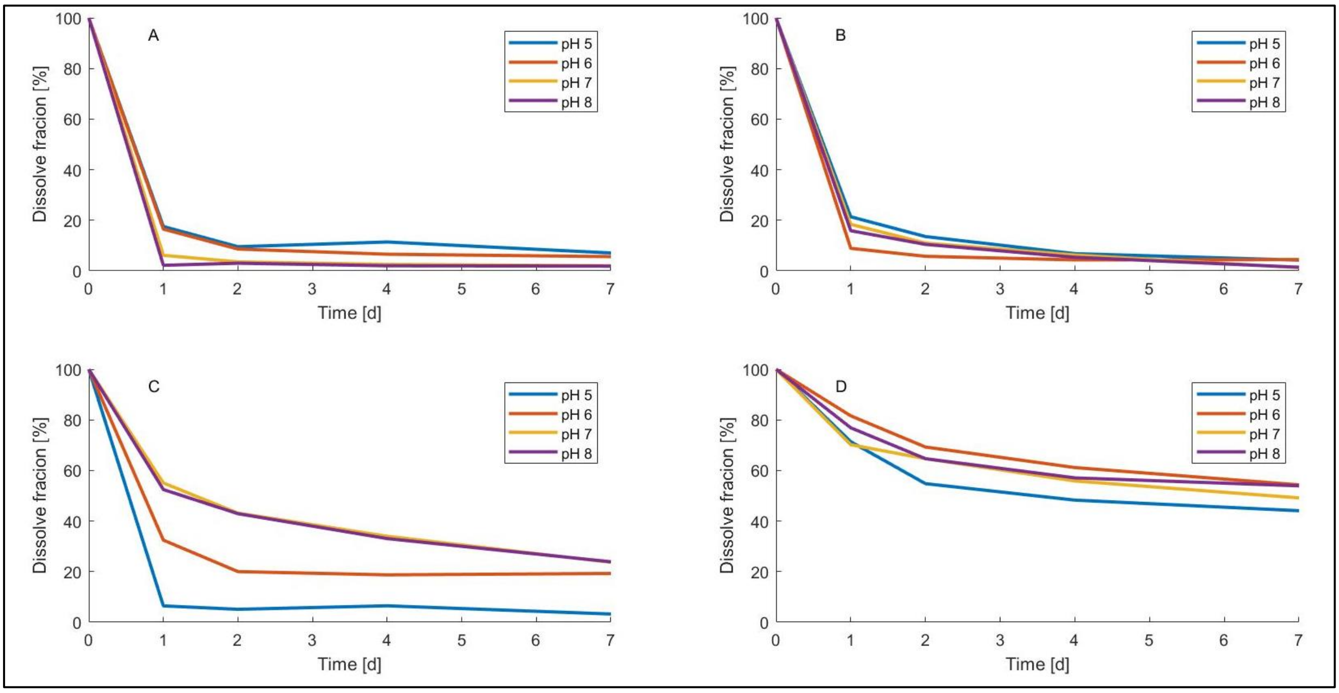

Figure S3. Adsorption dynamics of Er in batch experiments under different pH values (5-8) and humic acid (HA) concentrations. (A) 50 mg L^{-1} HA, (B) 20 mg L^{-1} HA, (C) 5 mg L^{-1} HA, (D) HA-free.

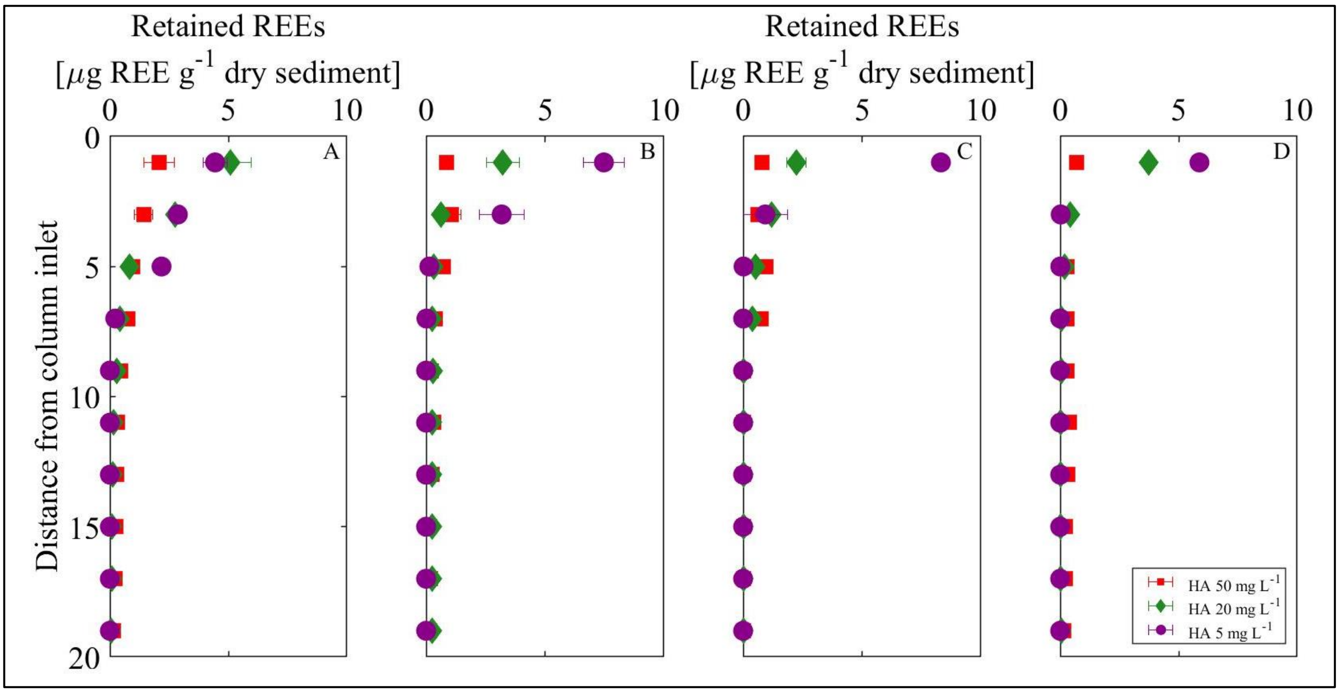

Figure S4. Vertical REE distribution profiles of La, Gd, and Er under different pH values (5-8) and HA concentrations (5, 20, 50 mg L^{-1}). (A) pH 5. (B) pH 6. (C) pH 7. (D) pH 8**.** An average of the three tested REEs (La, Gd, Er) is shown.

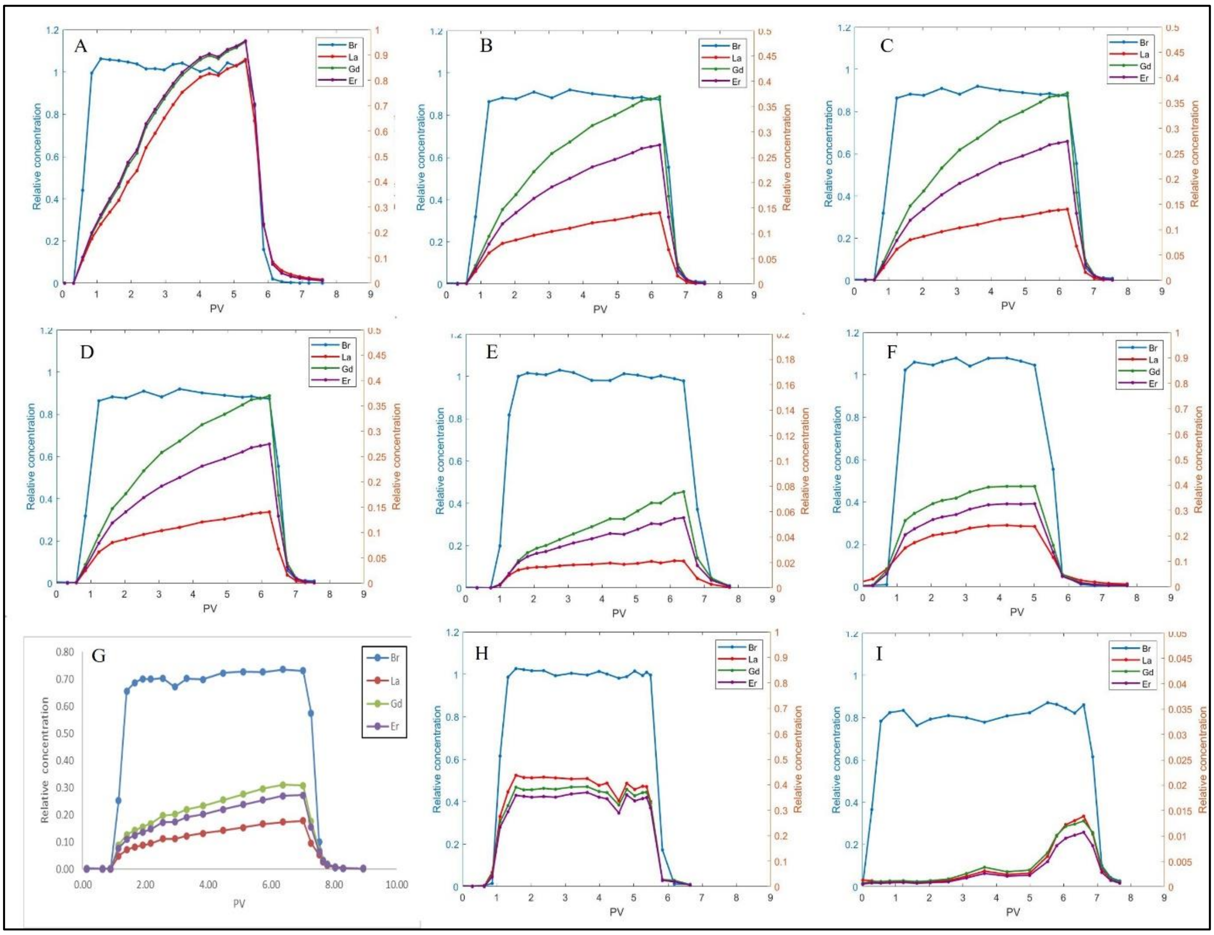

Figure S5. Column breakthrough curves replicate of La, Gd, Er, and Br tracer in different humic acid concentrations and pH solutions. Left y-axis: Br tracer relative concentrations. Right y-axis: REE relative concentrations. 50 mg L^{-1} HA: (A) pH 5, (B) pH 6, (C) pH 7, (D) pH 8, 20 mg L^{-1} HA: (E) pH 5, (F) pH 6, (G) pH 7, (H) pH 8, and 5 mg L^{-1} HA: (I) pH 8. Blue lines: Br- tracer, Red lines: La, Green lines: Gd, Purple lines: Er. Note: different y-axis maximum values.

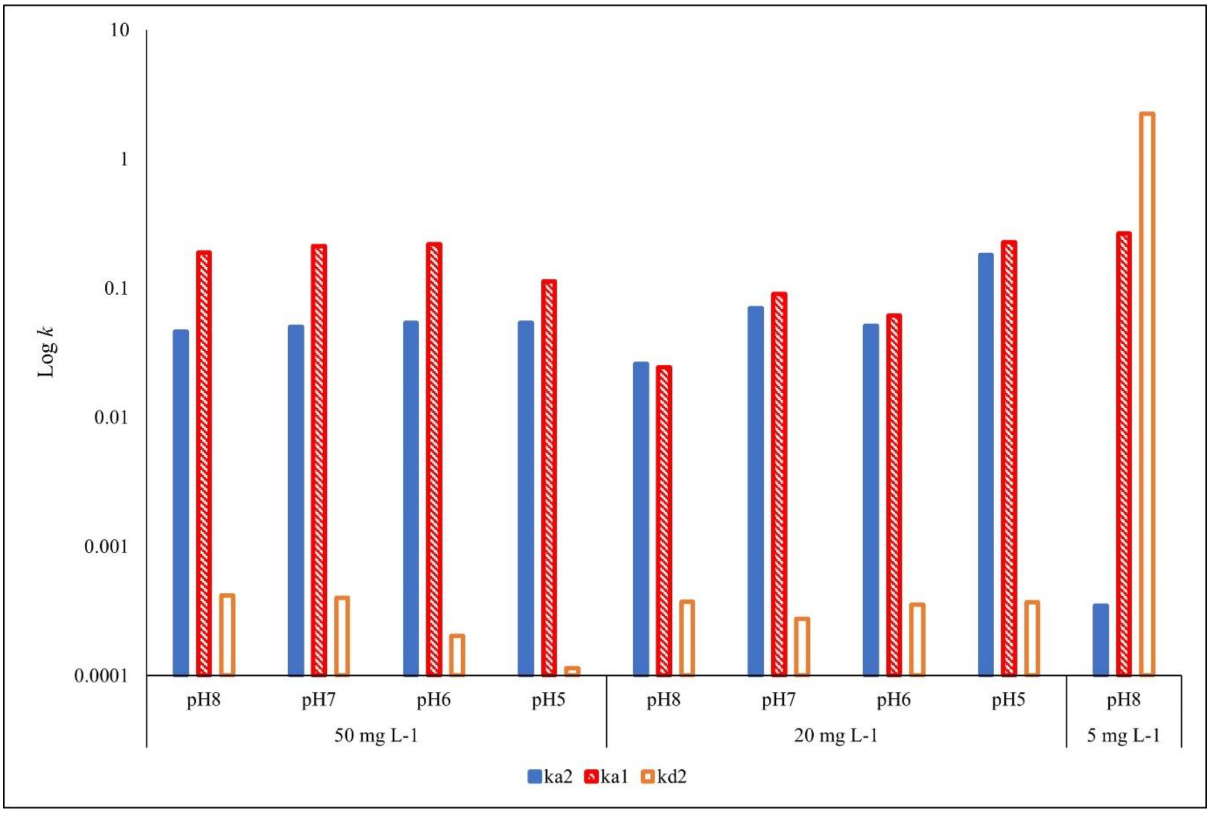

Figure S6. Model parameters for a two-site kinetic model of Gd breakthrough curve with attachment/detachment mechanisms, as k_{a1} is the attachment coefficient of site 1, and k_{a2} is the attachment coefficient of site 2. Parameters are presented for systems with humic acid concentrations of 5, 20, and 50 mg L^{-1} at pH 5-8.

REFERENCES

(1) Šimůnek, J.; van Genuchten, M. T. Modeling Nonequilibrium Flow and Transport Processes Using HYDRUS. Vadose Zo. J. **2008**, 7 (2), 782. https://doi.org/10.2136/vzj2007.0074.
